# The challenge of artemisinin resistance can only be met by eliminating *Plasmodium falciparum* malaria across the Greater Mekong subregion

**DOI:** 10.1186/1475-2875-13-286

**Published:** 2014-07-27

**Authors:** Cara Smith Gueye, Gretchen Newby, Jimee Hwang, Allison A Phillips, Maxine Whittaker, John R MacArthur, Roly D Gosling, Richard GA Feachem

**Affiliations:** 1 Malaria Elimination Initiative, Global Health Group, University of California, San Francisco, USA; 2 United States Centers for Disease Control & Prevention, Atlanta, USA; 3 The School of Population Health, University of Queensland, Brisbane, Australia

## Abstract

Artemisinin-based combinations are currently the most effective anti-malarials and, in addition to vector control, have led to significant declines in malaria morbidity and mortality. However, foci of artemisinin drug resistance have been identified in the Greater Mekong subregion (GMS) of the Asia Pacific, threatening the major gains made in malaria control and potentially creating a parasite pool that is more difficult to treat and eliminate. Efforts are underway to halt the spread of artemisinin resistance, including coordination of activities and funding, and identification of areas of suspected artemisinin resistance, now using a newly identified molecular marker. However, targeting resources to the containment of resistant parasites is likely inefficient and monitoring impact is challenging. A more sustainable solution is the rapid elimination of all *Plasmodium falciparum* parasites from the GMS. This strategy is more efficient for several reasons. First, a subregional strategy is in line with current commitment to elimination and will build upon the existing national political support for elimination as well as enhancing collaboration among countries. Second, the challenge of human mobility in the GMS is subregional in scope and requires a harmonized elimination strategy. Third, countries will need to improve and intensify malaria operations to reach elimination, and this will be a singular goal across the subregion. Rallying around the goal of *P. falciparum* elimination will not only utilize existing regional bodies to catalyze political and funding support, but will also leverage the funding already in place to achieve this subregional goal.

## Background

The Greater Mekong subregion (GMS) of the Asia Pacific, which includes the countries of Cambodia, China (Yunnan Province), Lao People’s Democratic Republic (PDR), Myanmar, Thailand, and Vietnam, is the epicentre of artemisinin resistance. Foci of resistance have been identified along the Thailand-Myanmar, Thailand-Cambodia, Vietnam-Cambodia, and Vietnam-Laos borders [1,2], and recently there has been a concerning report of resistance emerging in sub-Saharan Africa, specifically Angola [3]. Artemisinin-based combination therapies (ACTs) are currently the most effective anti-malarials [1], and, in conjunction with vector control, have led to the significant reduction of malaria morbidity and mortality worldwide. The continued development and spread of artemisinin resistance threaten these gains [4,5].

Resistance to anti-malarials has historically originated on the Thailand-Cambodia border; *Plasmodium falciparum* parasites resistant to chloroquine, then sulphadoxine-pyrimethamine, and finally mefloquine were first detected in this part of the world [5]. Initial signs of chloroquine resistance appeared in the late 1950s in Southeast Asia and spread across South Asia to East Africa by 1978 [3], then subsequently across the continent, leading to catastrophic increases in child morbidity and mortality in sub-Saharan Africa [1]. Artemisinin resistance could follow the same trajectory, leading to a persistent parasite pool that is harder to eliminate and can increase the incidence of severe or prolonged illness and mortality, particularly in low-transmission areas with reduced population immunity to malaria [6]. Given this history and the devastating potential impact of global artemisinin resistance, the new reports of the spread of resistance are of grave concern.

## Current approach

Efforts are underway to contain the spread of artemisinin-resistant *P. falciparum*, guided by the framework of the World Health Organization’s *Emergency Response to Artemisinin Resistance in the Greater Mekong Subregion (ERAR)*[1], which outlines the actions needed for improved coordination of activities and funding across the GMS. However, ERAR is in its nascent state and has had limited success to date. Attempts to identify areas of suspected artemisinin resistance through traditional drug efficacy sentinel site monitoring (e.g. monitoring slide positivity on day 3 post-treatment) have been small in scale and difficult to implement, although this process may become easier with the newly identified molecular marker associated with artemisinin resistance [7]. Even so, if identification of areas with resistance is challenging, monitoring the impact of resource-intensive containment efforts and successfully eliminating resistant parasites in these areas will be even more difficult. Moreover, the constant movement of people and parasites in this part of the world translates to continually shifting populations at risk and the ongoing potential to spread resistant infections beyond the area of containment.

In light of these challenges, operationally targeting resources to only those areas with resistant parasites has not been effective. Instead, targeting all areas with *P. falciparum* malaria transmission, regardless of whether resistance has been detected, negates the need to define drug resistance in all sites in real-time, and protects against the spread of resistance to new areas because transmission everywhere is reduced. Thus, there is a strong argument to be made that the only effective and sustainable way to respond to the current resistance situation is the rapid elimination of all *P. falciparum* parasites from the GMS.

## Recommended strategy: subregional *Plasmodium falciparum* elimination

### Political and financial commitment

**Figure 1:**
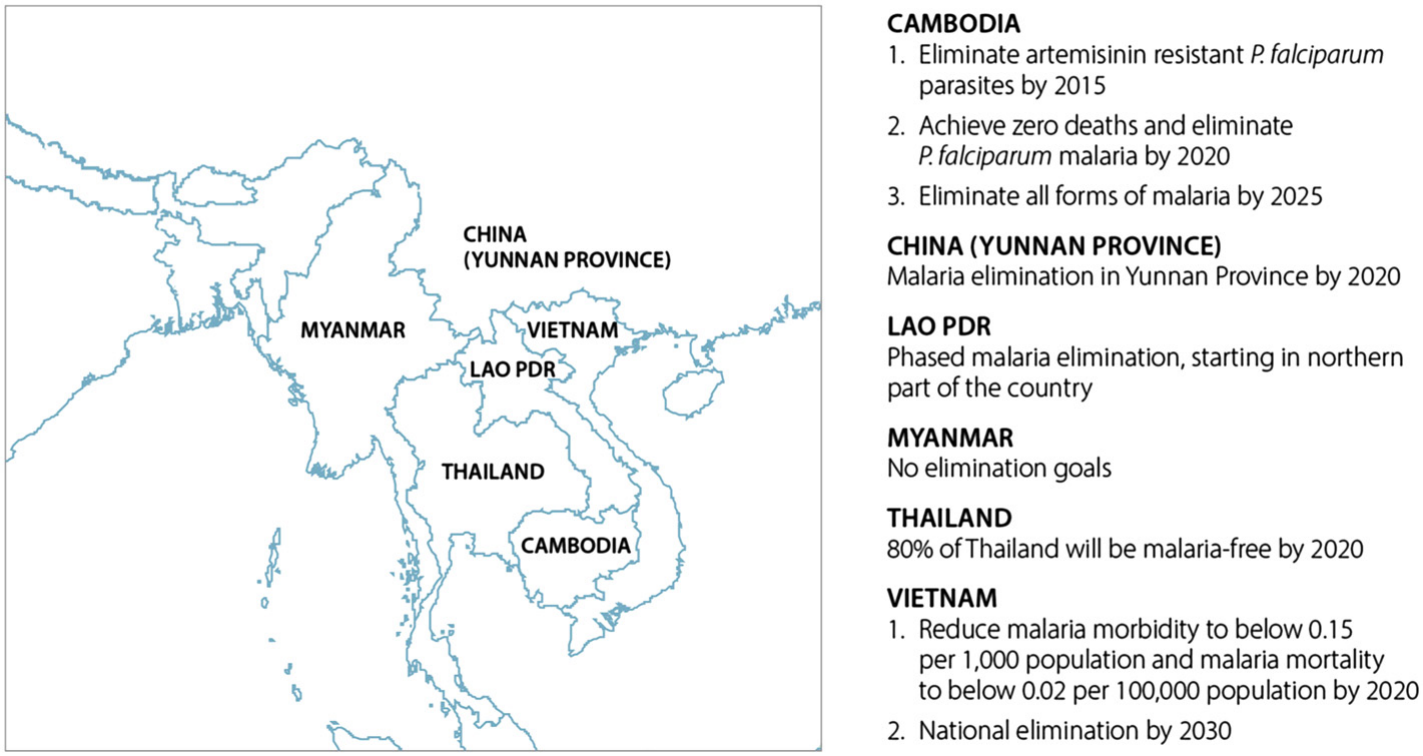
Countries in the Greater Mekong subregion and their elimination goals.

A subregional elimination strategy is in line with the current commitment to malaria elimination in the GMS and will build political support and enhance collaboration across countries. Five countries in the GMS have already declared an intention to eliminate all forms of malaria within their borders (Figure 1). Cambodia has defined and is actively working toward a *P. falciparum*-specific elimination goal, and four countries—Cambodia, China, Thailand, and Vietnam—have made major strides toward national elimination of all malaria species [8].

Political commitment for malaria elimination in the GMS is considerable, facilitated by five of the countries’ partnership with, and active participation in, the Asia Pacific Malaria Elimination Network (APMEN), a network of 15 countries across the Asia Pacific region that have declared goals of malaria elimination [8,9]. Leveraging this commitment, a GMS *P. falciparum* elimination initiative would bring together national programmes and partners around a single, strategic, time-limited goal, presenting an opportunity to achieve a regional public good. A focused, subregional effort specific to artemisinin resistance would improve coordination and harmonization of activities and funding across the six countries and multitude of partners, through the increase of communication and information sharing, launch of operational collaboration in border areas, and identifying and implementing a joint research agenda.

All of the countries of the GMS have intensified their control and elimination efforts and are, or will soon be, receiving technical and financial support to boost their operational capacities. Many technical partners are working on research and implementation in the region. Some countries, such as China and Thailand, have made major progress in their capacity for response while others, such as Myanmar, have some work to do to bring the malaria program to a level necessary to meet the resistance challenge. However, well-drafted plans for intensified *P. falciparum* control and elimination activities will not be impactful unless the GMS countries have the financial resources to make those plans a reality. Funding is now available for GMS countries to increase their capacity, a major source being the Global Fund grant to the Regional Artemisinin Initiative (RAI) [10], but additional and more coordinated funding is necessary to ensure sustainability throughout the region. Making the investment case for a *P. falciparum-*free GMS may spark the interest of other funders in a way that containment efforts thus far have not.

### Mobile populations

A subregional effort for elimination meets the challenge of human mobility in the GMS. The populations most at risk for development of resistance are primarily located in relatively underdeveloped and remote border regions. The lack of accessible services leads to inadequate detection of resistant malaria and low coverage of preventive and curative services. National malaria strategies typically focus on at-risk populations within country borders, but this approach fails to address the regional nature of migration patterns. Population movement through remote, forested areas and across porous borders increases the difficulty of accessing these groups by any one country. Thus, a comprehensive subregional strategy that relies upon international coordination is essential.

Large-scale regional infrastructure developments are underway that necessitate rapid action to prevent the spread of drug-resistant malaria. In late 2015, the Association of Southeast Asian Nations (ASEAN) will launch the ASEAN Economic Community (AEC). The AEC aims to reduce economic disparities among countries through the development of an integrated regional economy, which will create a freer flow of goods, services, capital, and labour, increasing human mobility in the region. Greater population movement in areas with confirmed resistance can facilitate its spread, and the risk will be compounded by the new transport corridors and infrastructure that will increase access to remote areas.

### Implementing subregional elimination

A GMS elimination goal requires that countries improve and intensify malaria control operations and management. A recent joint assessment of containment efforts found insufficient intensity, coverage and quality in the delivery of malaria control interventions [11]. Regional level guidance and technical assistance will help countries develop and maintain the more robust, sustainable and comprehensive set of strategies required to achieve *P. falciparum* elimination. Such strategies include early diagnosis and treatment, active case detection, case investigation, and effective measures to reduce and prevent transmission. These strategies should be implemented on a national level with robust regional guidance. Execution in a small, foci-based manner will not be as beneficial to the overall health system nor the malaria program. China’s new 1-3-7 malaria surveillance approach provides one operational model of surveillance and response; more countries in the region need to implement surveillance and response measures as an intervention. 1-3-7 defines actions and timelines for effective surveillance activities: case reporting within one day, case confirmation and investigation within three days and appropriate public health response to prevent further transmission within seven days [12]. National malaria programmes must also have adequate budgets for management to ensure that all activities are efficient, well-executed and well-supervised [1]. In addition, because the stakes are high, programmes must be equipped to measure and demonstrate impact.

## Conclusion

Rallying around an urgent and ambitious subregional *P. falciparum* elimination goal is the only way to effectively remove the historic epicentre of anti-malarial drug resistance. The newly formed Asia Pacific Leaders Malaria Alliance (APLMA) presents a timely opportunity: one way to signal commitment to a subregional elimination goal is to have members of the Alliance engage with public and private sector development partners in generating support and mobilizing resources. ASEAN is chaired by Myanmar this year, and encouraging ASEAN to formally indicate that improvements in regional economic trade are linked with anti-malarial drug resistance and subregional malaria elimination would accelerate elimination efforts. This is particularly true given the potential impact of the AEC on the spread of resistance.

Finally, proven malaria strategies with the explicit goal of *P. falciparum* elimination throughout the subregion must be aggressively implemented, without limiting their scope to already-identified resistant focal areas or populations. Progress towards this subregional goal is underway, with encouraging steps taken through the establishment of the RAI, funded by the Global Fund, the Regional Malaria and Other Communicable Disease Threats Trust Fund hosted by the Asian Development Bank [13], contributions to the ERAR from Australia, the Bill & Melinda Gates Foundation, and the US Government’s contribution to the President’s Malaria Initiative in the GMS. However, more concerted and long-running efforts are needed.

## Abbreviations

AEC: ASEAN Economic Community; ASEAN: Association of Southeast Asian Nations; APLMA: Asia Pacific Leaders Malaria Alliance; APMEN: Asia Pacific Malaria Elimination Network; ERAR: Emergency response to artemisinin resistance in the Greater Mekong subregion; GMS: Greater Mekong subregion; PDR: Lao People’s Democratic Republic; RAI: Regional Artemisinin Initiative; WHO: World Health Organization.

## Competing interests

The Global Health Group at UCSF exists in part to support global, regional and country efforts to achieve evidence-based malaria elimination. CSG, RG and MW are part of the APMEN Joint-Secretariat and RGAF is co-Chair of APMEN.

## Authors’ contributions

CSG, GN and JH conducted research and analysis and wrote the manuscript. AAP, MW, JM, RDG, and RGAF provided comments on drafts and contributed to the final manuscript. All authors read and approved the final manuscript.

## References

[B1] WHO Emergency response to artemisinin resistance in the Greater Mekong subregion: regional framework for action 2013–2015 2013 Geneva: World Health Organization

[B2] President’s Malaria Initiative Greater Mekong Sub-region Malaria Operational Plan FY 2014 http://www.pmi.gov/docs/default-source/default-document-library/malaria-operational-plans/fy14/mekong_mop_fy14.pdf?sfvrsn=12

[B3] Van Hong N Amambua-Ngwa A Tuan NQ Cuong DD Giang NTH Van Dung N Tinh TT Van Tien N Phuc BQ Duong TT Rosanas-Urgell A D’Alessandro U Van Geertruyden J-P Erhart A Severe malaria not responsive to artemisinin derivatives in man returning from Angola to Vietnam Emerg Infect Dis 2014 20 doi:10.3201/eid2007.140155 10.3201/eid2007.140155 PMC4073848 24963881

[B4] Dondorp AM Nosten F Yi P Das D Phyo AP Tarning J Lwin KM Ariey F Hanpithakpong W Lee SJ Ringwald P Silamut K Imwong M Chotivanich K Lim P Herdman T An SS Yeung S Singhasivanon P Day NPJ Lindegardh N Socheat D White NJ Artemisinin resistance in *Plasmodium falciparum* malaria N Engl J Med 2009 361 455 467 10.1056/NEJMoa0808859 19641202 PMC3495232

[B5] WHO Global report on antimalarial drug efficacy and drug resistance: 2000–2010 2010 Geneva: World Health Organization

[B6] Bjorkman A Bhattarai A Public health impact of drug resistant *Plasmodium falciparum* malaria Acta Trop 2005 94 163 169 10.1016/j.actatropica.2005.04.015 15893289

[B7] Ariey F Witkowski B Amaratunga C Beghain J Langlois A-C Khim N Kim S Duru V Bouchier C Ma L Lim P Leang R Duong S Sreng S Suon S Chuor CM Bout DM Menard S Rogers WO Genton B Fandeur T Miotto O Ringwald P Le Bras J Berry A Barale J-C Fairhurst RM Benoit-Vical F Mercereau-Puijalon O Menard D A molecular marker of artemisinin-resistant *Plasmodium falciparum* malaria Nature 2014 505 50 55 24352242 10.1038/nature12876 PMC5007947

[B8] Asia Pacfic Malaria Elimination Network 2014 http://www.apmen.org

[B9] Gosling RD Whittaker M Smith Gueye C Fullman N Baquilod M Kusriastuti R Feachem RGA Malaria elimination gaining ground in the Asia Pacific Malar J 2012 11 346 10.1186/1475-2875-11-346 23078536 PMC3504559

[B10] The Global Fund to Fight AIDS, Tuberculosis and Malaria Multicountry East Asia and Pacific (RAI) 2014 http://portfolio.theglobalfund.org/en/Country/Index/MER 10.1684/mst.2014.0309 24681635

[B11] Joint Assessment of the Response to Artemisinin Resistance in the Greater Mekong Sub-Region Summary Report 2012 Commissioned by Australian Agency for International Development, Department for International Development (UK), US Agency for International Development and the Bill & Melinda Gates Foundation, in collaboration with World Health Organization http://malaria2012conference.com/cms/wp-content/uploads/2012/10/Summary-of-the-Joint-Assessment-of-the-Response-to-Artemisinin-Resistance.pdf

[B12] Cao J Sturrock HJW Cotter C Zhou S Zhou H Liu Y Tang L Gosling RD Feachem RGA Gao Q Communicating and monitoring surveillance and response activities for malaria elimination: China’s “1-3-7” strategy PLoS Med 2014 11 e1001642 10.1371/journal.pmed.1001642 24824170 PMC4019513

[B13] Asian Development Bank Regional strategic response to malaria and other communicable diseases in Asia and the Pacific 2014 http://www.adb.org/projects/47272-001/main

